# Analysing successful public spaces in an urban street network using data from the social networks Foursquare and Twitter

**DOI:** 10.1007/s41109-016-0014-z

**Published:** 2016-11-16

**Authors:** Taras Agryzkov, Pablo Martí, Almudena Nolasco-Cirugeda, Leticia Serrano-Estrada, Leandro Tortosa, José F. Vicent

**Affiliations:** 1 Department of Computer Science, University of Alicante, Ap. Correos 99, Alicante, E-03080 Spain; 2 Department of Urbanism, University of Alicante, Ap. Correos 99, Alicante, E-03080 Spain

**Keywords:** Street network, Network analysis, Foursquare, Twitter, Data visualization

## Abstract

This paper analyzes success public spaces (specifically *plazas*) in the urban fabric of the city of Murcia, Spain. Two approaches were adopted. Firstly, the city was visualized as a complex network whose nodes represent *plazas*. A centrality algorithm was applied to determine the importance of each node. Secondly, data sets were used from social networks Foursquare and Twitter, which provide different types of data as well as user profiles. Foursquare data indicates user preferences of urban public spaces, while in this respect Twitter offers less specific user generated data. Both perspectives have facilitated two rankings based on the most visited plazas in the city. The results enabled a comparative study to determine the potential differences or similarities between both approaches.

## Introduction

The city is a complex system where a large amount of information and data are generated and used to determine its characteristics. The source of this vast information can be very diverse. Originally, the information was gathered from field work. More recently, data is available through Web services, social networks and other existing databases (open or protected).

Social media services generate huge quantities of data on a daily basis. In recent years the use of social media in service delivery has increased significantly. This in turn is constantly producing data for both research and commercial purposes.

In general, social media can offer visions on diverse aspects of social, economic, and political urban life, reflecting the different social network user profiles and their interests (Hong 2015). Research has shown that social network analysis measures can be useful to predict interesting urban phenomena. For example, to understand: the importance of particular junctions in transportation networks (Garrison 1960; Kansky 1963), the connectedness of rooms inside buildings (Casalania and Rittel 1967; March and Steadman 1971)), the flow of pedestrian traffic on city streets (Hillier 1996); and the distribution of retail and service establishments in urban areas (Porta et al. 2006; Sevtsuk 2010). As geo-referenced data are increasingly available on a global scale, new and readily accessible tools are needed to provide network analysis for interested parties across disciplines.

Social networks such as Facebook, Foursquare and Twitter are considered as the newest data sources (Adnan et al. 2014; Gantz and Reinsel 2011): ”a growing shift in internet browsing from PCs to mobile devices -tablets and smartphones"- (Mazzoccola 2014). Thus, as an activity that happens in the real world is shared on line, its location -latitude and longitude values- gets shared as well as part of the physical place’s digital overlay (López 2012).

This paper focuses on two of the most relevant social networks, Foursquare and Twitter. Twitter is a very popular social-networking and micro-blogging service which was launched in 2006, and within nine years had 300 million registered users worldwide, sending an average of 340 million tweets per day. This is a huge data source and the analysis of these data can provide useful insights into the places and situations in which users engage. Users can publish their opinions, ideas, stories, and news in messages that are up to 140 characters long.

Some restrictions are worth noting. For example, up to 1000 total updates on a Twitter account can be published per day from all the user’s devices. Twitter limits direct messages to 250 total per day on all devices. Only up to 150 application programming interface (API) requests to Twitter can be made per hour. In other words, each time a user performs an action on Twitter, an API request is counted. Up to 2000 people can be followed on Twitter.

The main objective of this paper is the analysis of successful public spaces in the urban network representing the downtown of the city of Murcia (Spain), by using data extracted from two social networks –Foursquare and Twitter– and comparing it to data gathered from fieldwork. Initially, the APA algorithm (Agryzkov et al. 2012) is applied to determine the importance of public places (*plazas* in the city) from the data collected through field work. In other words, with the application of the APA algorithm, which produces a centrality measure, we obtain a classification of the public spaces according to their importance in the network; so, we establish a ranking. Finally, we extracted and analyzed the data sets provided by the social networks Foursquare and Twitter, especially those related to the outdoors and recreation category where the word *plaza* appears. This way, taking both analysis we can make comparisons between them looking for possible similarities.

## The area of the city of Murcia studied and its *Plazas*

**Fig. 1:**
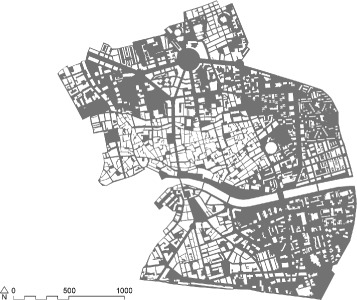
Map of the area studied of the city of Murcia. We take the historic centre of the city, which is the most important part from the point of view of commercial and leisure activities

Murcia is a Spanish city and capital of the Murcia autonomous community. It is located in the southeast of the Iberian Peninsula on the river Segura. Murcia is the seventh largest municipality in Spain, with a population of 441,354 inhabitants. The area of study is restricted to the historic centre of Murcia and its surrounding neighbourhoods. Its extension (in Fig. 1) is 40 hectares, characterized by a dense concentration of commercial venues and facilities, and thus, it is the most active area of the city.

In order to study the urban layout, we first represented it by an abstraction model. To represent the abstract model we used an urban street network (Crucitti et al. 2006a; 2006b). We created the network (urban street network) from a connected graph where the streets become undirected edges. Nodes usually represented the intersections of the streets, but we also assigned nodes to some points of interest in long streets. The urban street network allowed us either to represent the topology of an urban fabric as well as to organize the geo-located data. Subsequently, we developed a mathematical model or algorithm to analyse the network.

**Fig. 2:**
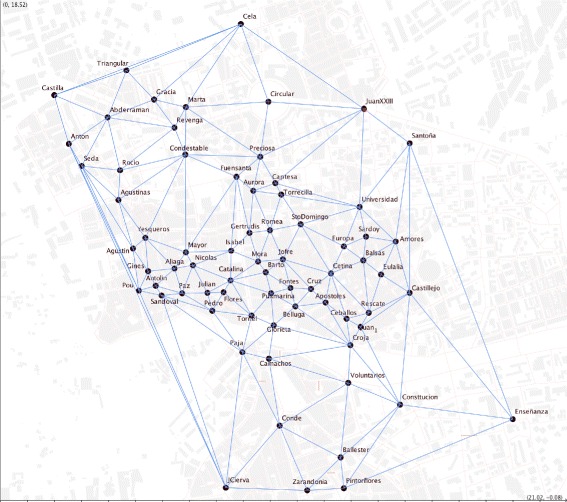
*Plazas* network. Here, we construct a network with the 72 *Plazas* existing in the urban street network of Murcia

Even though the geometric topological representation of urban street networks is often done as a primal graph where nodes are street intersections, the approach taken by this study is rather different. Highly visited public spaces -plazas- are taken as graph nodes (72 geographical points in total) using Delaunay’s triangulation as shown in Fig. 2. In other words, the socially relevant plazas of the city are the nodes of the city’s graph.

## Some data sets from different sources

Diverse data sources that provided different data sets have been used in this study. We analysed them to understand their essential characteristics. According to a study based on Instituto Nacional de Estadística INE statistics prepared by the National Observatory for Telecommunications, 18.6 million people access the internet periodically. Young people between 16 and 24 are the most frequent users (96 *%*), while the 65 to 75 years age group is that with less frequent users (25 *%*). The main online activities are: reading emails; information searches about products or services; and interacting through social networks.

The Sixth Annual Study of Social Networks presented by IAB Spain in February 2015 shows the evolution of social network penetration in Spain: it has increased from 51 *%* to 81 *%* of internet users from 2009 to 2015. The most used social networks according to this study among people aged over 18 years are, Facebook, Twitter and YouTube have recognition rates of 99 *%*, 94 *%* and 94 *%*, respectively. In the case of Facebook, the most renowned social network, it is the most “used or accessed”, with 96 *%*, followed by YouTube (66 *%*) and Twitter (56 *%*).

### Field work in the city of Murcia

In this section we describe the data source representing the information from the physical context of the city. The field work consists of a visual inspection of services and facilities related to tertiary activity located in the area of the case study. All services located in the first two floors of buildings, which are the areas exposed to public view from the street, were registered.

Therefore, we can say that the data collection process is a field study that consists of collecting the data or information we want to analyse or visualize. Subsequently, these data must be assigned to the nodes of the network so that each node has a set of numerical values associated with the information that is being studied. Essentially, the process followed to assign the information to the nodes was based on a computational geometry algorithm to count points inside irregular polygons.

The first objective was to study the city from the point of view of the facilities and its commercial activity. Therefore, all the information collected and geo-referenced was subdivided according to commercial sectors. So, in this field work analysis, we distinguish the following types of facilities: Type I Bars, restaurants, coffee, snack bar, (food-service). Type II Small shops. Type III Bank offices. Type IV Big shops (Department stores, malls, supermarkets,...).

The number of tertiary activities that have been collected through field work are: 552 venues (Type I), 2216 venues (Type II), 285 venues (Type III) and 33 venues (Type IV). Note the large number of activities of Type II (small shops) that have been collected and geo-located.

### Foursquare’ data set from the city of Murcia

Among social networks, Foursquare is a useful reference for identifying recommendations about local stores, restaurants, malls, or other activities in the city. Foursquare (foursquare.com) is, as categorized by Sui and Goodchild (Sui and Goodchild 2011), a social check-in site that enables users to share their whereabouts with their friends (Reed 2014) and, in most cases, with any on line user. The ”basis of the platform consists of user-generated venues for business and points of interests” (Reed 2014) from where Foursquare users can check-in. Currently, the number of registered individuals and businesses that are part of the Foursquare community surpasses the 50 million and 1.9 million businesses respectively (Foursquare, 2014). Additionally, the significant amount of the geographic information generated overtime on Foursquare is accessible to the public through Application Programming Interfaces (API) (Roick and Heuser 2013).

The selection of Murcia as a case study is based on the fact that Murcia is Spain’s fourth city in terms of the amount of activity on Foursquare (www.puromarketing.com/16/15391/comousan-espanoles-foursquare.html). Thus, data from this social network is analysed. According to the data downloaded, Foursquare categorizes each venue into five predefined categories: Outdoors & Recreation, Shops and Services, Food, Nightlife and Arts & Entertainment. In turn, each category is divided into a number of subcategories.

Since we are interested in the public spaces and, in particular, in the *plazas* of the city, we focus on the data extracted for the category Outdoors-recreation and on the subcategory *Plaza.* Table 1 shows data concerning Foursquare’s 20 *plazas* that have registered most visits in the geographical area under study. Note that 37 venues have been identified in the Foursquare data with the word Plaza in its subcategory. The table shows the number of visits, the number of check-ins as well as associated photos. Data reflected do not correspond to a particular time period but represent historical values since the venue’s original registration in Foursquare. Thus, both the number of visits and check-ins refer to the accumulated values since the venue’s existence on the network.

**Table 1:** Data from field work and Foursquare related to the subcategory *Plaza*

Plaza	Fieldwork data				Foursquare data			
	Type I	Type II	Type III	Type IV	Total	Visits	Check-in	Pictures
de las Flores	8	4	1	0	13	1219	2669	187
St. Domingo	12	6	0	0	13	788	2756	146
de la Catedral	5	2	1	0	8	650	1436	242
Circular	4	16	7	1	28	329	1589	46
Condestable	3	3	6	0	12	294	574	38
Sta. Isabel	4	15	6	0	25	233	787	22
Julian Romea	5	4	0	0	9	224	1169	52
Mayor	6	3	0	0	9	140	460	15
Sta Catalina	7	3	0	0	10	100	308	15
Europa	4	9	2	0	15	94	229	3
Díez de Revenga	3	15	5	0	23	90	644	16
Camachos	6	10	0	0	16	75	514	17
de la Merced	1	3	1	0	5	75	167	4
Cetina	3	10	1	0	14	63	451	11
San Juan	4	2	0	0	6	43	103	2
Juan XXIII	4	8	0	1	13	36	208	2
de la Cruz	0	0	0	0	0	36	60	12
Castilla	1	6	1	0	8	35	323	14
de los Apostoles	5	4	0	0	9	32	49	10
De la Seda	0	8	0	0	8	32	134	17

### Twitter’s data set from the city of Murcia

The Social Media Family carried out a study that concluded on December 31, 2014 about users’ profiles in Twitter and Facebook. It analysed the 50 most populated Spanish cities with the greatest presence on these social networks.

See (http://es.wikipedia.org/wiki/Anexo:Municipios_de_Espa%25C3%25b1a_por_poblaci%25C3%25b3n).

The findings highlight the following points relating to the use of Twitter and Facebook in Spain and in Murcia:

- Facebook users: Madrid ranked the first position with 3 million user profiles. Murcia ranked the 11th position with 260,000 user profiles.
- Twitter users: Madrid ranked the first position with 537,611 user profiles. Murcia ranked the 10th position with 73,943 user profiles.
- Footballers and football clubs are the most followed profiles. Singers and actors are the next most popular profiles.
- The most populated cities coincide with those that have a higher number of users registered on Facebook. However, this does not hold true for Twitter, where towns like Córdoba, León, Granada and Alicante have high numbers of users despite not being the most populated cities in Spain.

The data set used for this study consists of the tweets that have been produced in the area of the city of Murcia described above, from which we were able to get their geolocation for the period 11 to 20 June 2016. Data ranging from 12 June (Sunday) to 19 June (Sunday) were used. Two things should be noted: data obtained for the 11 June and 21 June are incomplete; and only geolocated tweets were used, and the remaining tweets stored in the database were discarded.

**Fig. 3:**
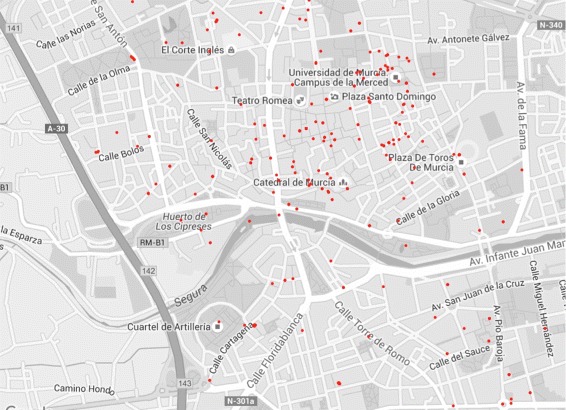
Tweets geolocated in the area of study during the day range from 11 and 19. We have geolocated tweets in the historic centre of Murcia during the day range from 11 to 19

The data was retrieved from a circled area with a radius of 4 km, whose centre corresponded to the geocentric coordinates of the city of Murcia, *c*(*l* *o* *n*,*l* *a* *t*)=(−1.1315,37.9830). Many geolocated tweets fell outside the area covered by this study. The data were stored in a table with the variables: day, hour, tweet identifier, longitude and latitude. A total of 1125 geolocated tweets were collected, 427 were outside the area of the city that we are analysing, which represents 38 *%* of all the tweets. The geolocated tweets obtained from the historic centre of Murcia are shown in Fig. 3. The topological arrangement of the tweets is particularly concentrated in areas around the cathedral and old town.

**Fig. 4:**
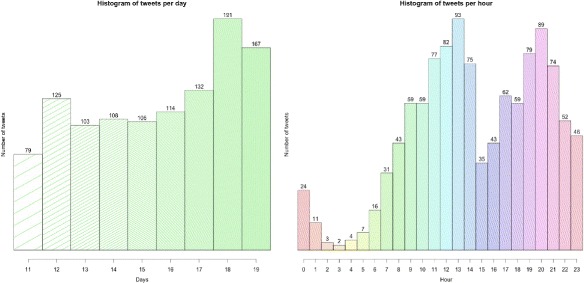
Tweets geolocated in the area of study during the day range from 11 to 19 per day and per hour. The image in the *left* shows the geolocated tweets in Murcia during the day range from 11 to 19, per day. The image in the *right* shows the frequency of tweets per hour in the same period of time

We conducted a small quantitative study of the frequency of tweets per day and per hour. The quantitative results are shown in Fig. 4. In the left image, on the *X* axis we have represented the days in which data were recorded and each bar reflects the frequency of tweets on that day, regardless of the time they were tweeted. Note that tweets from 11 June start at 11 am. Unsurprisingly, the days with a higher frequency of tweets are June 18 (Saturday) and June 19 (Sunday).

In Fig. 4 (right), the histogram indicates the number of tweets per hour, regardless of the day they have been tweeted. The decline in the number of tweets during the early hours is notable, especially from 1 am to 7 am, from which time the curve starts to rise.

Another observation is the existence of two time periods of peak activity. The first is in the morning, from 11 am to 14 pm, while the second occurs from 7 pm to 9 pm. The time frame that has a maximum value of tweets is 12 noon to 2 pm and 8 pm to 9 pm, just when the workday ends for commerce and shops. Also noteworthy is the significant decline in tweets during the hours after lunch, between 3 pm and 4 pm. The time after the lunch break, commonly known in Spain as the siesta makes this overall number of tweets fall significantly.

## The Adapted PageRank Algorithm as a centrality measure

The PageRank method (Page et al. 1999) was proposed to compute a ranking for every Web page based on the graph of the Web. In fact, PageRank constitutes a global ranking of all Web pages, regardless of their content, and based solely on their location in the Web’s graph structure. The purpose of the method is obtaining a vector, called PageRank vector, which indicates the relative importance of the pages. Since the calculation of the vector is based on the structure of the Web connections, it is independent from the person’s search request.

In (Agryzkov et al. 2012), Agryzkov et al. propose an adaptation of the PageRank model to establish a ranking of nodes in an urban network, considering the influence of external activities. In the following, we refer to this algorithm as the Adapted Pagerank Algorithm (APA algorithm). Although the APA algorithm has been applied to urban networks, it could be applied to other networks, whenever we analyze or represent additional information from the network by assigning numerical values to the different nodes of the network, (Berkhin 2005; Langville and Mayer 2005).

The central idea behind the APA algorithm for ranking the nodes is the construction of a data matrix *D*, which allows us to represent numerically the information of the network that we are going to analyze and measure. The algorithm proposed in (Agryzkov et al. 2012) is:

**APA algorithm.** Let us assume that we have a primal graph representing an urban street network with *n* nodes representing streets intersections. We proceed as follows:

- Obtain the transition matrix *A* from the graph of the network.
- Consider the different characteristics *k*_*i*_ associated to each of the nodes for the problem studied; evaluate them in each node. With these data, we construct the matrix *D* (data matrix).
- Construct a vector $\vec {v_{0}}$, according to the importance of each of the characteristics evaluated. This vector represents a multiplicative factor.
- Obtain a vector $\vec {v}$ by multiplying $D \vec {v_{0}} = \vec {v}$.
- Normalize $\vec {v} \rightarrow \vec {v^{*}}$, using the maximum norm.
- Construct the matrix *V*, from $\vec {v^{*}}$.
- Construct the matrix *M* ^′^=(1−*α*)*A*+*α* *V*, from *A* and *V*.
- Compute the eigenvector associated to the eigenvalue 1 for the matrix *M* ^′^. That is the ranking vector.

Summarizing, we can say that the mean feature of this algorithm is the construction of the matrix *D* and the vector $\vec {v_{0}}$. Firstly, the matrix *D* allows us to represent numerically the information we want to study; secondly, the vector $\vec {v_{0}}$ allows us to establish the importance of each of the factors or characteristics that have been measured by means of *D*.

In other words, we could say that the algorithm constitutes a model to establish a ranking of nodes in a network, with the objective to assigns a numerical value to each node according to its significance.

## Numerical results

The first objective of the paper is to apply the APA algorithm to the network generated by the *plazas* of the city. First, we proceed by determining all the squares (*plazas*) that are in the urban street network. We have identified a total of 72 *plazas* in the city area for study. These venues are shown in Fig. 2, where we identify the different *plazas* over the map of the city and construct a graph taking these venues as nodes and edges connecting neighbor *plazas*.

The data used for the algorithm launch are the data obtained from fieldwork, according to the four categories listed related to the commercial activity, where each of them is associated to a different business sector.

Note that the data collected by fieldwork are located in the entire urban network, i.e., the data do not distinguish any specific part of the network; therefore, they are not referred to the *plazas* that we are studying. Consequently, it was necessary to conduct a preliminary extraction process of the data corresponding to the nodes which form the network of *plazas* which are the subject of this study.

Table 1 reflects in detail the data concerning the venues identified in the urban fabric as *plazas*. The data shown in Table 1 correspond to the data of field work in four categories (type I to IV), respect to the venues *plazas* that have received more visits in the social network Foursquare. This table only includes data from the 20 *plazas* in the urban area studied that have received more visits from social network users.

The APA Algorithm was applied to the data collected for all the *plazas* that have been detected in the map of the city (there were 72). After running the APA Algorithm a classification of the nodes (*plazas*, in this case) was obtained according to their importance in the network. So, a ranking of the *plazas* of the city was established according to their importance in the network. In this study, the data are related to the commercial activity developed in the city, classified by different sectors. To see the details of the model used to perform the visualization of the ranking in the network, see (Agryzkov et al. 2012).

**Fig. 5:**
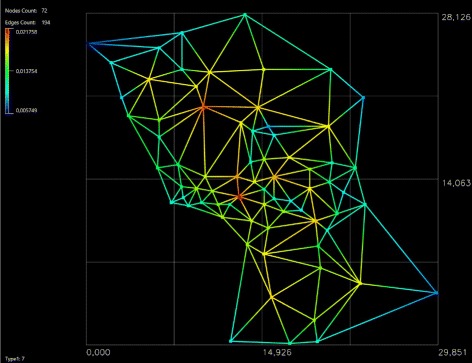
Visualization of the APA theoretical scheme over the graph of the *plazas*. Application of the APA centrality algorithm to the network of *plazas* in Murcia. Nodes in *red* colour are more important than others, while nodes in *blue* colour are less important in the network

Figure 5 represents a visualization of the network of *plazas* after running the APA algorithm. According to the chromatic colour scale used, red means highest importance while blue means lowest importance.

A Delaunay triangulation was used to build the graph in Fig. 2. For the rest of the study, the original topology of the triangulation was kept (Fig. 5) but excluding its initial geometric proprieties and the graph edges which fell beyond the urban area studied. The idea behind this was to keep the original topology of the plazas network. The transformation of the graph resulted in a concave network border (Fig. 5).

Consequently, we have two distinct scales: first, the scale of the domain of values that provides quantification of information and, on the other hand, the scale that provides us with the graphic scale. It is necessary to enhance a linear interpolation to set the colour that is assigned to each of the nodes, according to the amount of information associated with it. Once we have this colour range in the nodes, a graphical representation of the edges follows the same format representation. Using this visualization model by means of a chromatic scale in the graph, the result obtained for the graph where the nodes are the *plazas* in the area studied is shown in Fig. 5. Notably, the most important nodes in the *plazas* network are located not only in the city centre but in its historic centre, where the network of streets has a classic ancient layout with narrow streets and blocks forming irregular polygons shapes.

There are two different rankings of importance: the classification given by the APA Algorithm taking the commercial data of a field work; and the preferences of Foursquare users. It is interesting that some of the places that occupy very high positions in the ranking given by the visitors of Foursquare, also occupy high positions in the ranking offered by the APA algorithm. It is important to note that within the various types of data that Foursquare offers, we established a classification according to the tastes of network users evidenced from their visits to each venue and not the check-in that accumulates. The reason is that one user can make various check-ins of a venue.

The node *Plaza de las Flores*, occupying the first position in the social network Foursquare ranking, also ranks first in the classification given by APA algorithm. This coincidence is a remarkable circumstance, the absolute coincidence in the importance given to this venue (plaza), from the point of view of the interests of users of social network and within the framework of the network topology and commercial facilities.

**Fig. 6:**
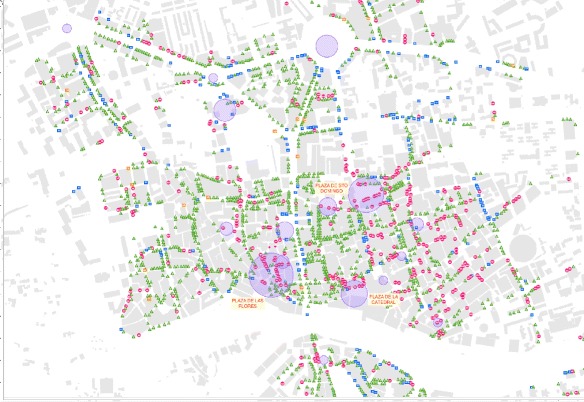
Most successful *plazas* over the geolocated facilities and venues collected by field work. A map where we show the most successful *Plazas* (circles) was superimposed to the endowments and venues related to field work. The radius of the circles is in direct proportion to its importance

Figure 6 hows a map of the historic centre of the city of Murcia with geolocations of all facilities and businesses that have been obtained in the fieldwork. Red represents the commercial food-service sector venues. In green are shops or small businesses, while in blue are the bank offices; finally, in orange, large department stores and supermarkets are represented.

Overlying this information the 20 most visited places by users have shown Foursquare. The circles have a size proportional to its importance. From Fig. 6 we highlight the correlation between the *plazas* that were most commonly visited by social network users and areas with a high density of commercial allocations or facilities. Especially significant is this coincidence in the three most visited *plazas* in Foursquare, where we found a remarkable concentration of commercial activity in both the venue itself and its surroundings.

**Fig. 7:**
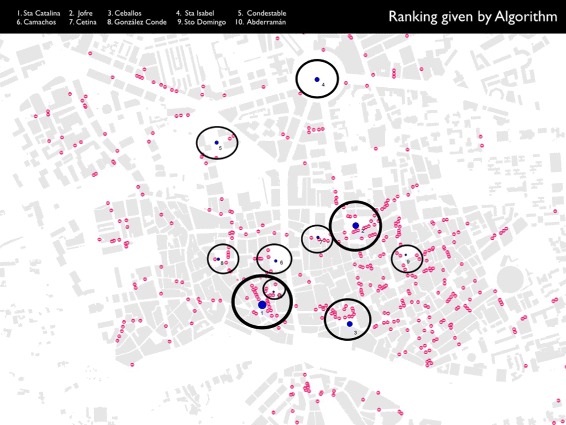
Most successful *plazas* over the field work data related to the food-service and leisure sector. A map where we show the most successful *Plazas* (circles) superimposed to the endowments and venues related only to the food-service commercial sector collected from the field work.The radius of the circles grows according to its greater importance

Figure 6 shows us all the allocations of field work relating to the four categories studied, i.e., the food-service sector (type I), small shops (Type II), bank offices and businesses (type III) and malls and supermarkets (type IV). More significant is this correlation if we only look at the food-service sector, where we have restaurants, bars, coffees, and so on. In Fig. 7 the *plazas* that succeed in accordance with the tastes or preferences of the social network users is directly related to a significant presence of facilities in the food-service commercial sector.

Regarding the data source Twitter, we generated a database from a set of requests made through the API, taking the epicenter of data as the tweets geolocated around the 3 most important *plazas* from the perspective of Foursquare data discussed above. Referencing the data in Table 1, the 3 *plazas* most visited by users of the social network are:

- *Plaza de las Flores*: *c*(*l* *o* *n*,*l* *a* *t*)=(−1.1353544,37.9844653).
- *Plaza de Sto. Domingo*: *c*(*l* *o* *n*,*l* *a* *t*)=(−1.129205,37.9870471).
- *Plaza de la Catedral*: *c*(*l* *o* *n*,*l* *a* *t*)=(−1.1280082,37.983871).

Consequently, we collected data from the geolocated tweets around these three points during the day range from 12 to 19. The data search was narrowed to 1 km away from the origin.

The searches have provided us with the following frequencies tweets:

- *Plaza de las Flores*: 583 tweets between day 12 to 19.
- *Plaza de Sto. Domingo*: 686 between day 12 to 19.
- *Plaza de la Catedral*: 648 between day 12 to 19.

**Fig. 8:**
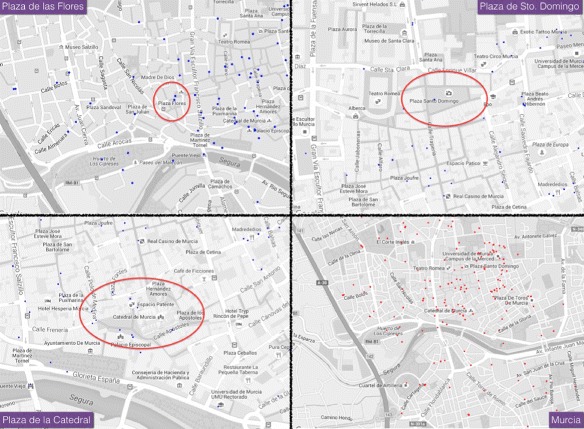
Geolocated tweets in Murcia between day 11 and 19. Image up left: Geolocated tweets around *Plaza de las Flores*. Image up right: Geolocated tweets around *Plaza de Sto. Domingo*. Image bottom *left*: Geolocated tweets around *Plaza de la Catedral*. Image bottom *right*: Tweets geolocated in the city

The location of the tweets is displayed in Fig. 8. In this figure we have four images. The first image (upper left) shows the geolocated tweets taking the node *Plaza de las Flores* as the central point. The second image (top right) shows the tweets and geolocation centered on the node *Plaza Santo Domingo*. The third image (lower left) shows tweets from around the node *Plaza de la Catedral*. The fourth image (bottom right) shows all geolocated tweets of the city of Murcia, including the extended area of study. All tweets are dated between day 12 and 19 June 2016.

Several observations can be made from the images shown in Fig. 8.

- The first three *plazas* in the ranking, obtained from analysing Foursquare data, do not have a concentration of tweets. This is particularly significant in the first two: the *Plaza de las Flores* and the *Plaza de Sto. Domingo*. Rather, the number of tweets is very low in these venues. In the cathedral and surrounding areas, the number of tweets increases, although not in a remarkable way. Unsurprisingly, in this area there are a number of venues related to food-service sector.
- The dispersion of the tweets in their geolocation is significant. The area of the city’s historic centre, near the cathedral and its surroundings, is very active. However, no great concentration exists at specific points. This dispersion is evident in the area of the *Plaza de Sto. Domingo*.
- Although a dispersion in the geolocation of tweets is observed, we can say that the urban area defined by the top three venues in the rankings studied represent the area where most geolocated tweets are collected. This is a consequence of the great commercial and leisure activity. Bars, restaurants, cafes and entertainment venues attract a large proportion of young people who are very active in social networks.

**Fig. 9:**
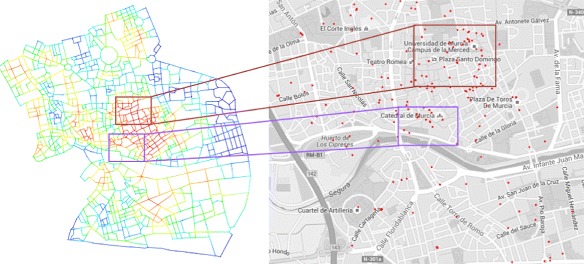
Visual comparison between geolocated tweets and human activity in the city. Image *left*: a map of the city visualizing the areas with more food-service and leisure activity. Image *right*: Geolocated tweets in the whole city

Figure 9 compares the geolocation of tweets during the study period of the most important areas within the city in terms of food-service and leisure sector. A correlation was observed between a high frequency of tweets and areas of high activity or flows of people.

A possible practical application of these results involves using the data to identify places in the city with greater commercial activity, which serves as a significant indicator of areas with a high presence of people, resulting in vibrant urban activity. Furthermore, awareness of these places can be very useful as reference points for tourists or people who do not know the city.

## Conclusions

We have studied the set of places that exist in the city center of Murcia from two perspectives. TThe first applies a classification algorithm of complex networks to determine the most important nodes. The second accesses data provided by Foursquare and Twitter users about their tastes and preferences in the city. The results show that the most visited venues (*plazas*) by both network users are plazas with a remarkable importance within the network, especially those which are at the top of the ranking in terms of the number of visits. Furthermore, data from these preferences suggest some fundamental characteristics of the city that can be confirmed by the theoretical study based on the classification algorithm. The urban activity taking place in the city centre is related to the food-service sector and to a lesser extent, to small retail outlets. This was crosschecked by comparing rankings given by the algorithm applied to the plazas network in order to display the network nodes and, thereby, the location of the most visited plazas by Foursquare users. Although the geolocation of tweets is much more dispersed, the visualization of their locations reinforces the idea of an existing urban area formed by the first three most important plazas where a high frequency of geolocated tweets have been collected.
